# Implementation of pharmacists' services into the care trajectory of older adults with neurocognitive disorder in multidisciplinary primary care clinics: A mixed-methods study

**DOI:** 10.1016/j.rcsop.2026.100786

**Published:** 2026-04-21

**Authors:** Dylan Bonnan, Edeltraut Kröger, Anne Maheu, Michèle Morin, Laurianne Bélanger, Isabelle Vedel, Machelle Wilchesky, Caroline Sirois, Clémence Dallaire, Étienne Durand, Yves Couturier, Nadia Sourial, Line Guénette

**Affiliations:** a Université Laval, Faculté de pharmacie, Canada; b Centre de recherche du CHU de Québec, Université Laval, axe Santé des populations et pratiques optimales en santé, Canada; c Centre d'excellence sur le vieillissement de Québec, Centre intégré universitaire de santé et de services sociaux de la Capitale-Nationale, Canada; d Centre intégré universitaire de santé et de services sociaux du Nord-de-l'Île-de-Montréal, Canada; e Université Laval, Faculté de médecine, Canada; f Centre intégré de santé et de services sociaux de Chaudière-Appalaches, Canada; g McGill University, Faculty of Medicine, Canada; h Donald Berman Maimonides Geriatric Centre, Canada; i Lady Davis Institute-Centre for Clinical Epidemiology, Canada; j Université Laval, Faculté des sciences infirmières, Canada; k Université de Sherbrooke, école de travail social, Canada; l Université de Montréal, école de santé publique, Canada

**Keywords:** Interprofessional primary care teams, Pharmacists, Implementation science, Neurocognitive disorders, Mixed-methods study

## Abstract

**Introduction:**

In Quebec, Canada, the “Alzheimer plan” encourages pharmacist participation in the care of older adults with neurocognitive disorders (NCDs) within multidisciplinary primary care clinics, known as Family Medicine Groups (FMGs). This study examined how these services were implemented from the perspective of physicians, nurses, and pharmacists.

**Methods:**

We conducted a convergent mixed-methods study combining cross-sectional questionnaires with semi-structured interviews across eight FMGs participating in a quasi-experimental project about the impact of pharmacists' activities on older adults' medication and well-being. Data collection and analysis were guided by the Normalization Process Theory and the Consolidated Framework for Implementation Research. A deductive thematic content analysis was carried out, using the five CFIR constructs as themes. Data from questionnaires were analyzed alongside interview data to gain a broader perspective on the implementation process.

**Results:**

Eighteen professionals (nine pharmacists, four physicians, five nurses) participated. From a quantitative perspective, pharmacists' integration was perceived positively: high agreement was reported regarding the intervention's potential value (90% pharmacists, 100% other professionals) and its adaptability (100% pharmacists, 80% other professionals). Also, 100% of physicians and nurses intended to continue to use pharmacists' services in the future. Qualitatively, facilitators included an easy-to-implement innovation (innovation characteristics), legislative changes (outer setting), the pharmacist's physical presence, strong interprofessional trust (inner setting), and proactive pharmacists' leadership (individuals) with regular team reminders (implementation process). Conversely, insufficient government funding (outer setting) and workload pressures were identified as primary barriers.

**Conclusions:**

Implementing pharmacist services for older adults with NCDs in FMGs is feasible and beneficial, improving workflow and team efficiency beyond NCD care. Strong interprofessional collaboration, effective communication, and clear role definition were key facilitators, while insufficient funding and workload pressures remain major challenges to implementation. Co-construction between primary care teams and decision-makers is essential to achieve successful scale-up.

## 1 Introduction

The pharmacist's scope of practice has considerably evolved over the last decades.^1^^,^^2^ In Quebec, Canada, pharmacists can adjust therapies, prescribe certain medications and laboratory tests.^3^ Since 2015, they have joined multidisciplinary primary care clinics, called Family Medicine Groups (FMGs), as independent non-dispensing professionals to support patients and colleagues with medication management.^4^ Within FMGs, they may also establish advanced practice partnership agreements with prescribers (i.e., general practitioners (GPs) and specialized nurse practitioners) to autonomously manage specific patient therapies under defined conditions.^5^

In 2019, Quebec launched the third phase of its *Alzheimer Plan,*^6^ shifting the care trajectory of older adults with neurocognitive disorders (NCDs) from specialized centers to primary care FMGs. This plan explicitly encouraged the pharmacists' participation, given the high prevalence of complex medication issues in this population, such as polypharmacy and potentially inappropriate prescribing.^7^ According to the recommendations of the Alzheimer Plan, these pharmacists are responsible for conducting medication reviews and for subsequent interventions or recommendations regarding the pharmacotherapy during the NCD diagnosis phase. They are also responsible, along with other healthcare professionals, for assessing medication efficacy and potential side effects at all follow-up visits for a person living with an NCD.

Pharmacists' contributions to patient outcomes are well documented,^8^ with evidence of improved adherence^9^ and disease control in conditions such as respiratory diseases^10^ and hypertension.^11^ However, the effect of pharmacist-led interventions in older adults in primary care remains uncertain, with mixed evidence regarding reductions in hospitalizations or mortality,^12^^,^^13^ and some evidence on improving the quality of medication of older adults with NCD.^14^ One of the factors that can influence the effectiveness of a pharmacist's service is its practical implementation in the settings and the willingness of professionals to adopt these new ways of working.^15^^,^^16^ Indeed, if a service does not fit with its environment or if the teams do not feel involved in its implementation, it may not produce the desired outcomes.^17^ It is also essential for a service to be adaptable to the unique realities of each environment to be effective.^18^ Several theories and frameworks have been developed to explore the implementation process of complex interventions or services in healthcare settings.^19^ These frameworks include factors from the settings, implementation processes, and individuals as key to the successful implementation of interventions.^16^

Since 2021, our team has been evaluating the impact of involving FMG pharmacists in the care trajectory of older adults undergoing evaluation for or living with NCDs. This broader project, detailed elsewhere,^20^^,^^21^ enabled us to conduct a mixed-methods sub-study among the nine FMGs that implemented the innovation (i.e., FMG pharmacists' involvement in the NCD care trajectory). We examined how clinical teams adapted or reconfigured this innovation to fit their organizational context and patient needs, as such adaptation may have a major impact on the intervention's effectiveness. Thus, the present study aimed to analyze the implementation of pharmacists' services within the NCD care trajectory from the perspective of the FMG professionals participating in the intervention group of the parent quasi-experimental study.

## 2 Methods

This study is reported in accordance with the Mixed Methods Reporting in Rehabilitation and Health Sciences (MMR-RHS) guidelines.^22^

### 2.1 Study design

To analyze the implementation of FMG pharmacists' services in the NCD care trajectory, we used a convergent mixed-methods approach.^23^ We combined a cross-sectional self-administered questionnaire with a multiple-case study using semi-structured interviews with FMG GPs, nurses, and pharmacists. Each FMG served as a case.

#### 2.1.1 Description of the care trajectory

Following Quebec's Alzheimer plan, the care trajectory began when a NCD was suspected by a GP, another healthcare professional, or when symptoms of cognitive impairment were reported by a patient or caregiver. The patients' GP referred them to an FMG nurse for a complete physical and cognitive assessment that included the Mini-Mental State Examination (MMSE) and/or the Montreal Cognitive Assessment (MoCA). Patients were then systematically referred to the FMG pharmacist for an evaluation, including a medication review and subsequent interventions to optimize the medication. In accordance with their scope of practice, pharmacists could adjust dosages, initiate or discontinue medications, and order laboratory tests.^3^ Although many actions could be performed autonomously, pharmacists also collaborated with the FMG team to align decisions with each patient's medical history and preferences.

#### 2.1.2 Setting and participants

Purposeful sampling was used to recruit participants from the intervention group of the parent study, i.e., those who had experienced FMG pharmacists' involvement in this care trajectory (the innovation). Pharmacists were first invited during research project meetings and by email. Each pharmacist then identified one collaborating general practitioner (GP) and one nurse, who were subsequently invited to participate by the research team. The participants completed the study questionnaire before their interviews.

#### 2.1.3 Questionnaire

We used the 23-item Normalization MeAsure Development (NoMAD)^24^ self-administered questionnaire, which assesses implementation processes from the perspective of professionals directly involved in implementing complex healthcare interventions. These items reflect the four constructs of Normalization Process Theory (NPT): 1) coherence (how people make sense of the new practice), 2) cognitive participation (commitment and engagement of individuals and teams), 3) collective action (how the new practice fits with existing workflow), and 4) reflexive monitoring (how actors appraise and understand the practice).

Before its use in this study, our team culturally adapted the French version of the NoMAD questionnaire to the Quebec pharmacy context.

We also complemented the NoMAD questionnaire with socio-demographic questions about the professionals (age, gender, profession, total experience in FMG, experience in current FMG) and the number of hours of work per week in the FMG. These variables were added since they may be influenced by, or influence, factors related to the implementation of the innovation.

#### 2.1.4 Semi-structured interviews

Semi-structured interviews explored how pharmacist services were integrated into the NCD trajectory. The interview guide was informed by the Consolidated Framework for Implementation Research (CFIR),^25^^,^^26^ which highlights five domains influencing implementation: innovation characteristics, outer setting, inner setting, individual characteristics, and implementation process. A draft guide was developed based on the literature and refined after a pilot interview with a pharmacist on the research team. This pilot interview was not included in the analysis.

Individual interviews were conducted in French between March and April 2024, three to four months after the intervention arm follow-up was completed in the parent study. A trained pharmacist-researcher (DB) conducted all interviews, 14 face-to-face and four via Microsoft Teams or phone. Interviews were audio-recorded and transcribed verbatim using noScribe,^27^ an Artificial Intelligence (AI)-based open-source software that runs locally without sending data to the cloud. The transcripts were reviewed in their entirety for accuracy following AI transcription.

The interview guide (Supplementary file 1) was adapted to address the role played in the innovation (e.g., questions specific to the vision of the pharmacist's role for GPs and nurses). The pharmacists and DB knew each other since the beginning of the broader project, which was coordinated by DB, but no relation between the GPs, the nurse and DB was established before the interviews.

All participants knew the objectives of the project in which the innovation was implemented and the goals of the interviews and questionnaires.

### 2.2 Analysis

In the transcribed verbatim, each participant was identified using an AX_Y code, where A is the profession (P = pharmacist, N=Nurse, G = GP), X is the unique identifier of each participant, and Y is the FMG identifier (e.g., P1_1 is the pharmacist number 1 working in the FMG number 1).

A deductive thematic content analysis, using both transcripts and field notes from the interviews, was conducted and discussed among team members throughout the process. We used the CFIR codebook template to integrate our results into the framework's domains and constructs, which served as the themes of our analysis. All CFIR constructs were included. First, two research team members (DB and CT) independently codified the transcripts from four interviews (20% of the total) to ensure code concordance and consistent use of themes. If any discrepancy remained after discussion, a senior member (LG) was available to resolve it. No new themes emerged during this phase beyond those already covered by the CFIR constructs. Second, DB processed the remaining interviews. The interviewees did not access the transcripts, but the results and their interpretation were discussed with them. Analyses were carried out using NVivo Software, version 14.

Quantitative data from the NoMAD questionnaire and socio-demographic questions were analyzed using descriptive statistics. Frequencies and percentages were used to describe categorical variables. For continuous variables, means were calculated. Given the descriptive objective of this implementation evaluation, no inferential statistics or hypothesis testing were performed.

The results were discussed with the rest of the team (LG, EK, CT and LB) to challenge assumptions and interpretations. The preliminary findings were presented to participating pharmacists to confirm accuracy and resonance with their experiences.

### 2.3 Data integration

The questionnaire results were triangulated with those from the semi-structured interviews. They helped confirm the results and develop a broader perspective on certain CFIR constructs. The results from the questionnaires and the interviews are presented and interpreted simultaneously in the results section. We used a joint display (available in Supplementary file 2) to present the integrated interpretations of both qualitative and quantitative data.

We used the CFIR constructs as our themes to present the results in the following sections. Answers to the questionnaires are presented in the theme sections most appropriate for joint analysis and interpretation.

For the sake of this article, quotes were translated from French into English using Microsoft Copilot AI services in the secure environment provided by Université Laval. The translations were reviewed by the authors for accuracy.

### 2.4 Ethical considerations

This research was approved by the Ethics Review Board of the *Centre intégré de santé et de services sociaux de Chaudière-Appalaches* (n°MP-23-2020-732). Participants were interviewed during their regular working hours and received no additional compensation.

## 3 Results

The characteristics of the 18 participants are presented in Table 1. Nine pharmacists, four physicians, and five nurses were interviewed, representing eight FMGs, from one urban (five FMGs) and one semi-rural (three FMGs) area. The reason for the non-participation was mostly a lack of time. Only two participants were men. The mean duration of the interviews was one hour per participant.

**Table 1:** Characteristics of participants (*n* = 18).

Participant	Age group (years)	Gender	Profession	Total experience in FMG (years)	Experience in current FMG (years)	Number of hours of work/week in the FMG
**FMGs from the urban area**						
**FMG #2**						
P2	40–49	Woman	Pharmacist	6–10	6–10	20
P3	≥60	Woman	Pharmacist	6–10	6–10	16
G1	<30	Man	G.P.	3–5	3–5	40
N1	40–49	Woman	Nurse	11–15	11–15	30
**FMG #3**						
P4	30–39	Woman	Pharmacist	3–5	3–5	3
**FMG #4**						
P5	40–49	Woman	Pharmacist	6–10	6–10	24
N2⁎	⁎	Woman	Nurse	⁎	6-10	⁎
**FMG #5**						
P6	30–39	Woman	Pharmacist	11–15	11–15	37.5
G2	≥60	Woman	G.P.	≥15	≥15	50
N3	<30	Woman	Nurse	1–2	1–2	34.5
**FMG #6**						
P7	30–39	Woman	Pharmacist	1–2	1–2	9
G3	<30	Woman	G.P.	3–5	3–5	6
**FMGs from the semi-rural area**						
**FMG #1**						
P1	30–39	Woman	Pharmacist	3–5	1–2	35
N5	50–59	Woman	Nurse	11–15	11–15	37.5
**FMG #7**						
P8	50–59	Man	Pharmacist	6–10	1–2	21
**FMG #8**						
P9	40–49	Woman	Pharmacist	6–10	6–10	35
G4	≥60	Woman	G.P.	≥15	11–15	45
N4	50–59	Woman	Nurse	11–15	11–15	37.5

FMG: Family Medicine Group.

G.P.: General Practitioner.

⁎ : Did not answer to the questionnaire.

Generally, pharmacists had fewer years of experience in FMGs than other professionals because their involvement in these settings was more recent. They were also more often working there part-time than other professionals: the average number of hours per week was 22 (compared with 35 for other professionals). The rest of the time, they continued to work in community pharmacies, hospitals, or long-term care settings.

**Fig. 1:**
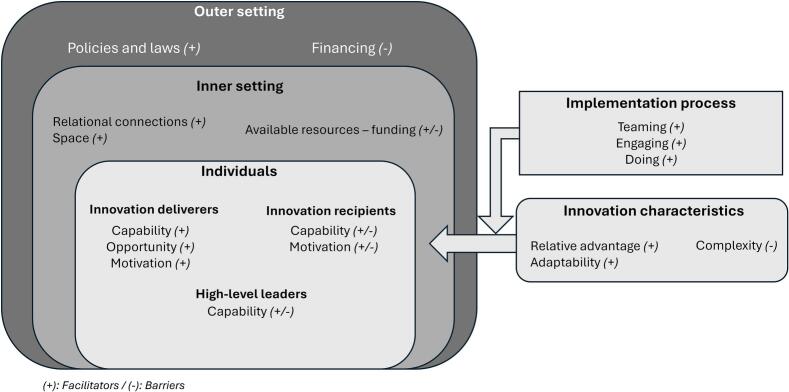
Structure of Consolidated Framework for Implementation Research themes and sub-themes influencing the implementation of pharmacist services in participating family medicine groups.

Fig. 1 provides a graphical representation of the identified CFIR themes and sub-themes.

### 3.1 Innovation characteristics

#### 3.1.1 Sub-theme relative advantage: The innovation presented a strong relative advantage according to all the professionals

**Fig. 2:**
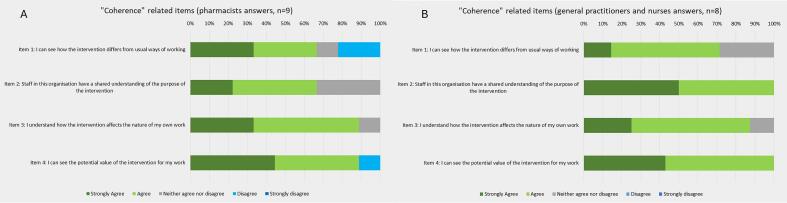
Results of the “Coherence”-related items from the Normalization MeAsure Development questionnaire.

Overall, nurses and GPs expressed a positive perception of pharmacists' involvement in the care trajectory of older adults with NCDs, recognizing its added value for their practice and older adults care (71% strongly agree or agree; Fig. 2B, item 1). Interviews confirmed that all three professional groups perceived a clear relative advantage of integrating pharmacists into the care trajectory. GPs highlighted that pharmacists helped optimize their time, enabling them to manage more patients despite an unchanged overall workload.

“*I think we save time... I think we save time because it allows me to do the things I had planned. The patients… we have a bit of a schedule, we would say we are going to do this, but the schedule has shortened, by doing this [involving the pharmacist], instead of taking years, we have saved time*.” G2_5.

#### 3.1.2 Sub-themes complexity and adaptability: The intervention was not complex to implement, even though the complexity of the intervention itself

Some pharmacists reported that the intervention had become fully embedded in their routine practice, blurring the distinction between the research project and usual care (25% disagree (*n* = 2). Fig. 2A, item 1).

However, some professionals noted the complexity of pharmacists' work, often linked to polypharmacy and the need for multiple interventions during the follow-up.

*“There are some [patients] with whom we worked on several interventions. (...) During the six months [of* *follow-up], there was still a lot of work to do*.” P3_2.

**Fig. 3:**
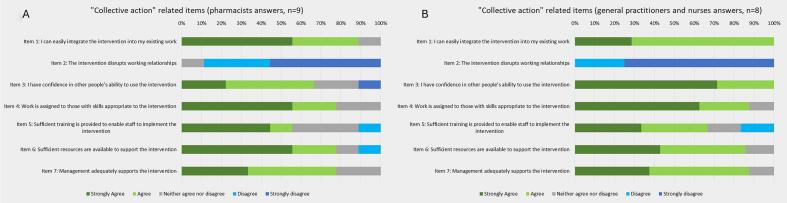
Results of the “Collective action”-related items from the Normalization MeAsure Development questionnaire.

Despite these challenges, GPs and nurses generally reported minimal constraints in involving pharmacists. Moreover, most professionals agreed that the new trajectory aligned well with their workflow (89% (*n* = 8) of pharmacists and 100% (n = 8) of GPs and nurses strongly agreed or agreed; Fig. 3A and B, item 1) and could be adapted to the organizational context of each FMG (e.g., scheduling appointments or organizing operations differently). The interviews confirm that the trajectory was easy to implement in their workflow.

### 3.2 Outer setting

#### 3.2.1 Sub-theme policies and laws: The modified laws governing pharmacy practice facilitated the pharmacists' interventions

External factors appeared to facilitate the implementation of pharmacist services within the NCD care trajectory. Legislative changes in Quebec pharmacy practice, such as expanded prescribing rights and the introduction of advanced practice partnership agreements, enabled pharmacists to optimize therapy more efficiently.

*“Fortunately, we have the [advanced practice partnership] agreement. Because before, the time I wasted was incredible. They [the GPs] were wasting their time, but I was wasting three times more. So, it allowed me to do a lot more volume in terms of interventions. And to waste less of my time, too*.” P6_5.

However, another participating pharmacist expressed difficulty with these agreements, finding them too engaging for a part-time practice. This participant chose not to use this option and instead worked in a more conventional manner with the GPs at her clinic.

“*I think the partnership agreement will only bring me more problems. For me, there is no logical advantage. I'm not there often enough.”* P5_4.

#### 3.2.2 Sub-theme financing: Government-provided funding was considered insufficient to support pharmacists' activities

Funding for pharmacist positions in FMGs was another recurring concern. Government allocations were considered insufficient to meet the needs. This is one of the explanations for the increased prevalence of part-time work among pharmacists, with pharmacists averaging 22 h per week. In some clinics, adjustments have been made to compensate for this issue, as discussed in the following section.

### 3.3 Inner setting

#### 3.3.1 Sub-themes relational connections and space: Interprofessional collaboration and pharmacist physical presence in the FMG are key enablers of the implementation of their activities

Several organizational factors within FMGs greatly facilitated the implementation of pharmacist services in the NCD care trajectory. Interprofessional collaboration emerged as a key enabler, supported by positive relationships among team members. Nurses and GPs emphasized the importance of teamwork and informal communication channels, such as “hallway conversations” or shared lunch discussions.

“*I think it's the collaboration of everyone that makes us follow the trajectory overall, and that's about it. I think it's the team's collaboration, in the end, that makes it a success.*” N5_1.

Integrating pharmacists into the NCD care trajectory did not negatively impact the existing working relationships among the healthcare team. According to the pharmacists, 89% (*n* = 8) either disagreed or strongly disagreed with the notion that their inclusion disrupted these relationships (Fig. 3A, item 2). Similarly, all of the GPs and nurses (100%, n = 8) strongly disagreed or disagreed with that idea (Fig. 3B, item 2).

In some FMGs, written protocols outlining the NCD trajectory and professional roles were accessible to all staff, fostering shared understanding and facilitating timely referrals (67% of pharmacists and 100% of GPs and nurses agreed or strongly agreed; Fig. 2A and B, item 2).

Physical presence of pharmacists within FMGs was another critical factor for collaboration and workflow integration, even when unrelated to NCD-specific activities.

*“As I say, just coming even if it's only once a week.* *‘Hello, you know, I'm developing this tool with this student. [...] Each time, I still slip an idea into their heads. I think the more contacts there are, the better it is in life.*” P5_4.

#### 3.3.2 Sub-themes available resources – funding: Additional resources from the FMGs facilitated implementation but are not a long-term solution

Limited funding from external sources constrained pharmacists' presence, as already mentioned. Some FMGs implemented compensatory measures, such as reallocating budgets from other professional positions or special projects, to increase pharmacist availability. Even if these measures were useful for implementing the trajectory in some FMGs, they were not a viable option for long-term maintenance.

*“They even asked, because when we went from 10 to 7 [hours per week], for us pharmacists, it was really a big problem. So, the GPs were super collaborative, and they decided to take on more of their GAP patients'* *forecast [time allowed by the ministry for patients without a regular GP]*.” P9_8.

### 3.4 Individuals

#### 3.4.1 Sub-themes innovation deliverers (capability and motivation): Professionals felt motivated and legitimate to take part in the intervention, but some personal traits could limit the required collaboration

**Fig. 4:**
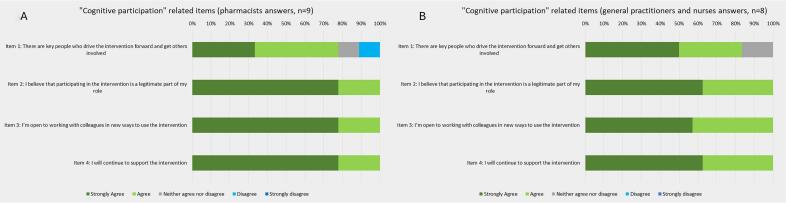
Results of the “Cognitive participation”-related items from the Normalization MeAsure Development questionnaire.

Professionals across FMGs demonstrated strong motivation to engage in the innovation. GPs and nurses actively referred patients to pharmacists, while pharmacists perceived their role as legitimate for optimizing care for older adults: 100% of pharmacists, GPs, and nurses agreed or strongly agreed that they were legitimate participants in the innovation (Fig. 4A and B, items 2) as well as their colleagues (Fig. 4A and B, items 2 and 3).

The interviews provided further insight into the role of each professional in the trajectory. Notably, GPs highlighted the practical benefits of pharmacist involvement, particularly for patients with complex medication regimens.

*“It's very useful to have someone, especially for patients who have a lot of medications and a heavy burden on that side.* *So, I find it very helpful to collaborate with a pharmacist.*” G3_6.

Individual attitudes and personality traits occasionally posed barriers to collaboration or to the adoption of new practice models. For example, two GPs in two different clinics reported that certain colleagues collaborated less with the pharmacist, primarily due to personal factors.

*“Some people, it's by default, sometimes, due to personality. There are people who can't work in a group. [...] who don't understand, who can't grasp the advantage of working in a group. [...] Some, it's because they're old like me, okay? And it's hard to change the mentality of someone old who has worked for years.*” G2_5.

They also highlighted that time and collaborative effort could partially mitigate these personal barriers. For example, when pharmacists showed their contribution in other cases or with other colleagues, it motivated reluctant GPs to get involved.

*“Older GPs, I have one who has been practicing here for 25 years. At first, he didn't refer much. [...] Since we allowed administrative staff to refer directly to pharmacists, I think they have seen more cases handled by the pharmacist; they saw ‘this is really useful*”. G1_2.

#### 3.4.2 Sub-theme innovation recipient (motivation): Patients' motivation to receive the intervention was generally positive, helped by the recommendation of GPs and nurses

Patient engagement was generally positive, though some reluctance was noted. GPs and nurses attributed this to the burden of additional appointments for patients and caregivers.

*“I talk to my pharmacists, [and] sometimes my pharmacists are surprised. It's that they end up seeing the patients who accept, [but] there are patients who refuse to see the pharmacist. [...] So, we have to be aware of this bias that not all patients want to see the pharmacist.*” G1_2.

On the other hand, some pharmacists reported that the GP's recommendation to schedule an appointment with the pharmacist was a strong motivator for several patients.

#### 3.4.3 Sub-theme high-level leaders (opportunity): Management support was generally sufficient to implement the intervention

Leadership support (FMG managers, GP in charge) was generally perceived as adequate (78% of pharmacists agreed or strongly agreed; Fig. 3A, item 7), though isolated cases of insufficient managerial engagement were reported.

*“I didn't get any support, that's all. [...] I thought that having the GPs there would be enough. But I think that... It's because before, we didn't really have a head GP...*” P4_3.

### 3.5 Implementation process

#### 3.5.1 Sub-themes teaming and engaging: Regular reminders and implication of nurses were key factors in the early stages of the implementation

Participants employed several strategies to integrate pharmacist services into the NCD care trajectory. Initially, the innovation was introduced during team meetings, accompanied by repeated reminders to maintain engagement.

Regular reminders were considered essential, as referral rates to the pharmacist services tended to decline without reinforcement. Once the initial introduction to the innovation was complete, this strategy appeared sufficient to sustain the implementation process.

*“Once we made a reminder, there was an increase in referrals. It would taper off, and we had to remind them again to see another increase in referrals from the nurses.*” G1_2.

Another strategy was to involve nurses more closely, as their role was central to the innovation's success. As the professional who performed cognitive tests and evaluations at the GPs' request, they were in the best position to refer patients to the pharmacist.

*“Training at the professional level, meeting with the nurse, especially. For me, the nurse is central.*” P5_4.

#### 3.5.2 Sub-theme engaging and doing: Pharmacists must actively promote the intervention for successful implementation

The successful implementation of the innovation also required the right attitude from the pharmacist, who had to be proactive within the team and bring the intervention to the attention of the rest of the staff.

*“The FMG pharmacist needs to be somewhat of the project leader. (...) And I think it also falls to this person to make reminders, check the adherence to the project by other professionals, and verify why, if it's having trouble getting started.*” N1_2.

#### 3.5.3 Sub-theme tailoring strategies: Tailoring strategies, such as stricter referral criteria, may be needed

**Fig. 5:**
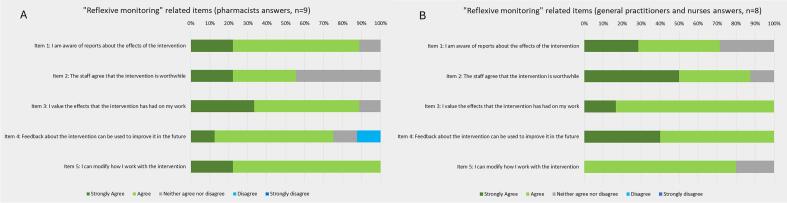
Results of the “Reflexive monitoring”-related items from the Normalization MeAsure Development questionnaire.

Globally, all professionals agreed that the intervention was adaptable to fit their own setting (100% of pharmacists and 80% of other professionals agreed; Fig. 5A and B, item 5). Notably, pharmacists systematically reviewed all patients undergoing cognitive assessment at the beginning of the project. However, high referral volumes led to delays, prompting adaptation through referral criteria based on medication type (e.g., antipsychotics, benzodiazepines), number (e.g., five or more), or specific comorbidities.

*“They would refer everyone to me, and I provided more [interventions], so I had delays in doing it. And when I got to do the evaluation, sometimes it was a bit too late. [...] So I think that often by looking at a pharmacological file, looking at lab results, looking at the type of patient, we can already tell who will benefit more versus less.*” P1_1.

### 3.6 Early sustainability and dissemination

Once established, the trajectory became routine for GPs and nurses, who expressed willingness to sustain it (100% agreed; Fig. 4B, item 4). Pharmacists also reported that their involvement in the NCD trajectory facilitated the development of other care trajectories and expanded their role in the FMG. For example, some pharmacists reported being involved in refractory pain or heart failure trajectories.

## 4 Discussion

In this study, we report the results of the implementation process of pharmacists' services within FMG primary care teams for older adults undergoing screening for or recently diagnosed with NCD using a convergent mixed-methods study. The qualitative and quantitative results were consistent and enabled further exploration of key aspects of the implementation process. No discrepancies were noted.

Overall, pharmacists, GPs, and nurses all felt that the pharmacist's services were valuable for both patients and the work organization. The new care trajectory was generally considered easy to implement and maintain over time. Globally, the pharmacists, GPs and nurses shared a similar vision on the benefits of the involvement of pharmacists in the NCD trajectory, according to both the interviews and the questionnaire. We did not observe significant differences in the implementation or in the views of participants according to their urban or rural location.

The success of this implementation was mainly due to factors related to the “inner settings” and “individuals” domains of the CFIR. The barriers we identified were mainly related to the “outer setting” or “individuals” CFIR domain, including the burden for some patients and their informal caregivers to see an additional healthcare provider, as well as insufficient pharmacists' time, according to the allowed funding from the Ministry of Health, to carry out all the required tasks. Importantly, according to the interviewees, when pharmacists are well established in their FMG, the benefits of their participation in the NCD care trajectory extend beyond patients with NCDs. Indeed, pharmacists reported becoming more involved with patients with other care needs for whom their expertise has been recognized, thanks to this project.

Factors linked to inner healthcare settings are well established in the literature as essential elements for the success of pharmaceutical services. Among these, good communication between the various members involved, particularly with GPs, is of utmost importance.^28^^,^^29^ To foster this collaboration, the pharmacist's physical presence in the FMGs was often cited as crucial in our study. Previous research has recognized that the availability of physical space is perceived as an element favouring collaboration through formal and informal exchanges in interprofessional primary care teams, as well as in FMGs with a pharmacist.^30^^,^^31^ Conversely, problems of communication with other professionals are a frequent barrier mentioned in settings such as community pharmacies,^32^^,^^33^ partly because of this lack of links with GPs.

Our findings indicate that the increased participation of pharmacists in the care trajectory has led to a more efficient workflow within the team, a trend that has also been observed in other studies.^34^^,^^35^ Good collaboration between the professionals and a clear definition of tasks for each of them are, however, a prerequisite.^34^^,^^35^ When these criteria are not met, tension and dissatisfaction can arise among the professionals involved.^36^ Our study also uncovered that some GPs could be reluctant to fully engage in increased collaboration with pharmacists. In a previous study on the greater involvement of Quebec pharmacists in long-term care homes,^37^^,^^38^ the researchers also encountered such barriers. To overcome these challenges, tools and training courses for physicians were developed and implemented. Although these strategies do not eliminate all barriers, they support physicians in navigating new collaborative models and dispel certain misconceptions about pharmacists' recent expanded scope of practice.

To encourage the patients and their caregivers to meet the FMG pharmacist, which can be perceived as an additional burden for some of them, it is important to rely on motivating factors for patients, such as patient-centered communication, a strong climate of trust within the FMG, and recognition of the pharmacist's additional expertise by the other professionals.^39^^,^^40^ In our study, the fact that the appointment with the pharmacist was discussed by the nurses and by the GPs certainly encouraged patients' participation. It highlighted the importance that other professionals placed on pharmacists' involvement in the NCD trajectory, mutual trust, and communication between the professionals.^41^ One must also address patients' personal barriers, such as the additional time needed for visits and transport, to facilitate access to the pharmacist's services and ensure the intervention's implementation success42., 43., 44 (e.g., flexible appointment dates, phone meetings, combining meetings with different professionals at the same time).

Finally, the financial barriers to successful innovation implementation that were encountered in our study were also observed in previous studies.^33^^,^^45^ Although various strategies have been developed in some FMGs to overcome financial barriers and thus increase pharmacists' working hours, these efforts were considered insufficient to meet the growing demands over time. To mitigate this issue, more stringent reference criteria were implemented in some settings and are viewed as a feasible option. For example, a threshold of five to eight chronic medications appears to be a relevant indicator of inappropriate polypharmacy. It may signal the need for pharmacists to involve the FMG pharmacist in the care trajectory of older adults with cognitive decline.^46^ Other useful indicators could include the use of potentially inappropriate medications such as benzodiazepines and antipsychotics.

## 5 Strengths and limitations

Our study has several strengths. First, employing two theoretical frameworks (CFIR and NPT through the NoMAD questionnaire) widely used in health services implementation research reinforces the robustness of our results. Indeed, the triangulation of results from semi-structured interviews exploring CFIR factors and the NoMAD questionnaire, which explores constructs of Normalization Process Theory, enabled a thorough description of the implementation process across different settings and highlighted the key elements present in most participating FMGs. Second, the diverse FMG environments, ranging from rural to urban areas, allowed us to consider a broad scope of realities perceived among various professionals in these settings, increasing our study's generalizability.

However, there are also certain limitations that must be acknowledged. In some FMGs, we did not meet all involved professionals, generally because of time constraints or because professionals felt they did not have the necessary experience with the innovation to answer our questions. This latter fact may indicate that the innovation was less well-established in these environments. Moreover, the FMGs that participated in this study were generally settings with positive work climates, i.e., environments with a strong culture of collaboration and where the innovation fitted their organizational values. As a result, we may have interviewed more motivated and involved professionals practicing in relatively favorable environments for such innovations. Therefore, one can expect to encounter more barriers, particularly those related to the inner setting and individuals' characteristics, when implementing the innovation in other FMGs. Finally, the prior relationship between the interviewer (DB) and some participants may have limited their willingness to discuss negative aspects of the implementation process. To avoid this, they were repeatedly assured that their responses would remain confidential and that no judgment would be made based on them. Conversely, this existing relationship may have encouraged them to be more open to discussing the issues they encountered during implementation.

## 6 Implications for practice

To ensure the successful implementation of this innovation in further multidisciplinary primary care clinics, it will be crucial to discuss, early on, the pharmacists' involvement. For instance, new care settings should develop clear procedures, explicitly define the role and responsibilities of each professional, and when pharmacists' time is limited, establish specific criteria to identify which patients would most benefit from their involvement. The pharmacist should act as the implementation leader, particularly in the first months, and must demonstrate good interpersonal and professional skills, as well as being open to interprofessional collaboration. Teaming up with other professionals, providing frequent reminders of the services, and involving nurses and administrative staff in the care trajectory are essential factors for the success of this innovation implementation.

Other key elements, such as mutual trust and efficient communication, are more challenging to establish, yet remain crucial for the successful implementation of a new care trajectory. These elements can be better integrated by supporting FMG teams during the early stages of adoption, notably through the involvement of local opinion leaders^47^ (e.g., experienced pharmacists, nurses and GPs with a lived experience of the innovation) who can provide one-on-one training or educational visits. Finally, financial considerations must not be overlooked, as the number of hours allocated to pharmacists within FMGs was considered insufficient to fulfill their mission completely and improve patient access to these services.

## 7 Conclusion

The implementation of pharmacists' services in Quebec multidisciplinary primary care clinics (Family medicine groups) for older adults undergoing cognitive assessment or living with NCD was generally positive. The GPs and nurses had a favorable opinion of the pharmacists' services and appreciated their added value. Good interpersonal skills and interprofessional collaboration, high-quality formal and informal communication, and a clear understanding of each other's roles in the care trajectory were key to a successful implementation. However, specific barriers must be addressed, such as inadequate funding of pharmacists' hours of service or potential challenges to collaboration. Decision-makers should consider these limitations and address them within co-construction processes with the primary care clinics' team to achieve a large-scale implementation of this service.

## Funding statement

This work was supported by the Fonds de Recherche du Québec (FRQ 2020-VILL-279547). During this work, D.B. received a doctoral training scholarship (#332830) from the FRQ – Santé (S). C.S. was the recipient of a FRQ-S Junior 2 research scholar bursary. Line Guénette is currently receiving a senior FRQ-S research scholar bursary.

## Consent to participate

Each professional gave audio-recorded verbal consent to participate in this study.

## Consent for publication

Not applicable.

## CRediT authorship contribution statement

**Dylan Bonnan:** Writing – review & editing, Writing – original draft, Visualization, Validation, Software, Resources, Project administration, Methodology, Investigation, Formal analysis, Data curation, Conceptualization. **Edeltraut Kröger:** Writing – review & editing, Visualization, Validation, Supervision, Resources, Methodology, Investigation, Funding acquisition, Conceptualization. **Anne Maheu:** Writing – review & editing, Visualization, Resources, Methodology, Funding acquisition, Data curation, Conceptualization. **Michèle Morin:** Writing – review & editing, Visualization, Methodology, Funding acquisition, Conceptualization. **Laurianne Bélanger:** Writing – review & editing, Software, Project administration, Investigation, Data curation. **Isabelle Vedel:** Writing – review & editing, Funding acquisition, Conceptualization. **Machelle Wilchesky:** Writing – review & editing, Funding acquisition, Conceptualization. **Caroline Sirois:** Writing – review & editing, Supervision, Methodology, Funding acquisition, Conceptualization. **Clémence Dallaire:** Writing – review & editing, Funding acquisition, Conceptualization. **Étienne Durand:** Writing – review & editing, Funding acquisition, Conceptualization. **Yves Couturier:** Writing – review & editing, Methodology, Funding acquisition, Conceptualization. **Nadia Sourial:** Writing – review & editing, Methodology, Funding acquisition, Conceptualization. **Line Guénette:** Writing – review & editing, Visualization, Validation, Supervision, Software, Resources, Project administration, Methodology, Investigation, Funding acquisition, Data curation, Conceptualization.

## Declaration of competing interest

The authors declare no potential conflicts of interest concerning this research, authorship and/or publication of this article.

## Appendix A Supplementary data

Supplementary material 1 Image 1

Supplementary material 2 Image 2
