# Supplementary material for: Implementation of pharmacists' services into the care trajectory of older adults with neurocognitive disorder in multidisciplinary primary care clinics: A mixed-methods study
Source: Explor Res Clin Soc Pharm. 2026 Apr 21;23:100786. doi: 10.1016/j.rcsop.2026.100786 (PMC13144580; doi:10.1016/j.rcsop.2026.100786)
Supplement: Supplementary file 1 — Supplementary material 1 [file mmc1.docx]

**Supplementary file 1. SEMI-STRUCTURED INDIVIDUAL INTERVIEW *GUIDE*****

***Translated in English using Microsoft Copilot for the sake of the article. The translations were reviewed by the authors for accuracy.*

**Introduction**

- Thank the participant for his/her time and for agreeing to participate.
- Discuss the objective of the interviews: to gather his/her opinions on his/her experience with the GPS [the name given to the intervention], project. The interview will explore several areas: his/her work environment and its influence, changes made to his/her way of working to implement GPS, relationships with other professionals, the future of the intervention over time...
- Inform him/her that there are no right or wrong answers and that the goal is simply to gather his/her opinions and experiences. He/She will not be judged on his/her responses, and the content of the interviews will remain confidential.

**QUESTIONS FOR PHARMACISTS**

**About GPS**

- **To begin, I would like you to introduce yourself and briefly describe your role within the FMG.**
- **What does the GPS intervention represent for you?**
  - *If possible, explore the "intervention" and "data collection and research" sections if the person does not mention them.*
- **Why did you want to participate in GPS?**
- **How was GPS implemented in your environment?**
  - *Changes in the way of working with other professionals. Discuss barriers and facilitators at this time, and support from other professionals.*
- **What impacts has GPS had on your daily life?**
  - *Way of working, impact on patients, and organization of the FMG.*

**Internal Environment and Individuals**

- **How has GPS changed your relationships with other professionals in the FMG?**
  - *Discuss the acceptance of recommendations, the more systematic use of the pharmacist’s services by professionals for cognitive disorders, as well as other types of patients.*
- **How do older adults with NCD perceive your role?**
  - *Also discuss their satisfaction with this new model and their expectations.*

**External Environment**

- **What external elements to the FMG have facilitated the implementation of GPS?**
  - *Discuss the role of the research team and monthly/clinical meetings (training), and the Alzheimer Plan.*
- **How has collaboration with community pharmacists evolved with GPS?**
  - *Changes in the way of collaborating, ensuring that interventions do not "collide" with each other.*
  - *Consideration of mutual interventions – transmission of information between each other.*
  - *Vision of the personal role in FMG vs. the role of the community pharmacist.*

**Sustaining the Intervention**

- **How will GPS continue in the environment?**
  - *Distinguish between pharmacist intervention and research.*
  - *Discuss modifications to be made or what will be preserved.*
- **Are there specific parts of the project that you would like to keep to continue the interventions?**
  - *Discuss clinical meetings and monthly meetings with other pharmacists.*

**Conclusion**

*•* **If you had recommendations to optimize the implementation of the GPS model in other FMGs, what would you say?**

- *Do you have any warnings regarding the implementation of the model in FMGs? If so, what are they?*
- *Is there anything else you would like to share or add regarding your experience with the new practice model?*

**QUESTIONS FOR GPs**

**About GPS**

- **To begin, I would like you to introduce yourself and briefly describe your role within the FMG.**
- **What does the GPS intervention represent for you?**
  - *Explore the "intervention" part and ask if they participate in patient data collection.*
- **Why did you agree for the FMG to participate in the GPS project?**
- **How was GPS implemented in your environment?**
  - *Changes in the way of working with other professionals. Discuss barriers and facilitators at this time.*

**Internal Environment and Individuals**

- **How has GPS changed your perception of the pharmacists working with you?**
  - *Discuss the acceptance of recommendations, the more systematic use of the pharmacist’s services by professionals for cognitive disorders, as well as other types of patients.*
- **Have you noticed a change in your workload since the involvement of the pharmacist in GPS?**
  - *Explore the time spent responding to recommendations, responding to pharmacists during their analyses, and the exchanges they have regarding patients.*
- **How would you describe the collaboration between all FMG professionals (nurses, pharmacists, social workers, you) since GPS regarding the management of NCD older adults?**
- **How have older adults with NCD reacted to this new way of working?**

**External Environment**

- **What external elements to the FMG have facilitated the implementation of GPS?**
  - *Discuss the role of the research team and monthly/clinical meetings, the Alzheimer Plan.*

**Sustaining the Intervention**

- **In your opinion, how will GPS continue in the environment?**
  - *Distinguish between pharmacist intervention and research.*
  - *Discuss modifications to be made or what will be preserved.*
- **Do you think you will continue to involve pharmacists in the management of your older adult patients with NCD? With other pathologies?**

**Conclusion**

- **If you had recommendations to optimize the implementation of the GPS model in other FMGs, what would you say?**
- *Do you have any warnings regarding the implementation of the model in FMG teams? If so, what are they?*
- *Is there anything else you would like to share or add regarding your experience with the new practice model?*

**QUESTIONS FOR NURSES**

**About GPS**

- **What does the GPS intervention represent for you?**
  - *If possible, explore the "intervention" and "data collection and research" sections if the person does not mention them.*
- **How was GPS implemented in your environment?**
  - *Changes in the way of working with other professionals. Discuss barriers and facilitators at this time.*

**Internal Environment and Individuals**

- **How has GPS changed your perception of the pharmacists working with you?**
  - *Discuss the acceptance of recommendations, the more systematic use of the pharmacist’s services by professionals for cognitive disorders, as well as other types of patients.*
- **Have you noticed a change in your relationship with pharmacists since the beginning of GPS?**
  - *Explore the use of the pharmacist’s services, exchanges on specific cases, and the general relationship. Also, explore how the pharmacist relies on the nurse for nursing-related issues.*
- **How would you describe the collaboration between all FMG professionals (doctors, pharmacists, social workers, you) since GPS regarding the management of NCD older adults?**
- **How have older adults with NCD reacted to this new way of working?**

**External Environment**

- **What external elements to the FMG have facilitated the implementation of GPS?**
  - *Discuss the role of the research team and monthly/clinical meetings, the Alzheimer Plan.*

**Sustaining the Intervention**

- **How will GPS continue in the environment?**
  - *Distinguish between pharmacists’ interventions and research.*
  - *Discuss modifications to be made or what will be preserved.*
- **Do you think you will continue to involve pharmacists in the management of older adults with NCD? With other pathologies?**
  - *Ask for their help more systematically on medications, have them perform analyses for patients...*

**Conclusion**

- **If you had recommendations to optimize the implementation of the GPS model in other FMG teams, what would you say?**
- *Do you have any warnings regarding the implementation of the model in FMG teams? If so, what are they?*
- *Is there anything else you would like to share or add regarding your experience with the new practice model?*
