# Supplementary material for: Implementation of pharmacists' services into the care trajectory of older adults with neurocognitive disorder in multidisciplinary primary care clinics: A mixed-methods study
Source: Explor Res Clin Soc Pharm. 2026 Apr 21;23:100786. doi: 10.1016/j.rcsop.2026.100786 (PMC13144580; doi:10.1016/j.rcsop.2026.100786)
Supplement: Supplementary file 2 — Supplementary material 2 [file mmc2.docx]

# Supplementary file 2. Joint display of qualitative and quantitative findings

| **Themes** | **Qualitative data**  (semi-structured interviews) | **Quantitative data** (NoMaD questionnaire) | **Integrated interpretations** |
| --- | --- | --- | --- |
| **Innovation characteristics** | | | |
| Relative advantage | Most professionals reported seeing a clear advantage of the integration of the pharmacist into their work.  Some pharmacists highlighted the fact that their participation in the trajectory is become routine. | - 90% of pharmacists and 100% of other professionals responded seeing the potential value of the intervention for their work. - 20% of pharmacists disagree that the intervention differs from usual ways of working. 30% of other professionals neither agree nor disagree. - 90% of pharmacists and 100% of other professionals value the effect of the intervention on their own work. - 55% of pharmacists and 100% of other professionals agreed that the staff believe the intervention is worthwhile. | The implication of pharmacists in the care trajectory offers a strong relative advantage for the professionals and their work.  In some cases, the innovation had already become the new standard, which explains why some professionals did not perceive the intervention’s added value or any difference from their usual ways of working. |
| Adaptability | The professionals could modify the trajectory to fit their need and their existing workflow. For instance, patients could be met by the pharmacist right after the nurse evaluation or on another day. Pharmacists could also perform the initial meeting by phone or in person, depending on the preference of the patients and their caregivers. | - 100% of pharmacists and 80% of other professionals agreed they can modify how they work with the intervention. | Even if not all professionals reported modifying the intervention to meet their needs, they all recognized how they could adapt it.  This adaptability facilitates the implementation of the pharmacists’ activities in each clinic, depending on their work organisation and preferences. |
| Complexity | Some pharmacists reported their actions could be difficult, depending on the patient’s profile. They sometimes have to perform multiple interventions in a few weeks.  However, the other professionals reported minimal constraints in involving the pharmacist. | - 90% of pharmacists and 100% of professionals agreed they can easily integrate the intervention into their existing work. | Overall, the professionals did not report major difficulties in integrating the pharmacist into the care trajectory. The only significant challenge reported by pharmacists was not directly related to the trajectory itself, but rather to the complexity of conducting medication reviews in a population with complex needs and frequent inappropriate polypharmacy. |
| **Inner setting** | | | |
| Relational connections | The professionals reported a good interprofessional collaboration in their clinics.  These connections could be formal (meetings to discuss a complex case) or informal (hallway conversations, lunch together).  In multiple clinics, these bonds existed before this integration of pharmacists into the care trajectory. | - 65% of pharmacists and 100% of other professionals agreed that the staff in the organisation have a shared understanding of the purpose of the intervention. - 90% of pharmacists and 100% of other professionals disagree that the intervention disrupts working relationships. More specifically, 55% of pharmacists and 75% of other professionals strongly disagreed with this item. - 55% of pharmacists and 100% of other professionals agreed that the staff believe the intervention is worthwhile. | The existence of informal connections seems to be an important marker of good team collaboration which favors the implementation of the pharmacist’s activities. These informal connections are facilitated by the pharmacist’s presence in the clinic and may lead to a positive perception of the intervention. |
| Available resources (funding, access to knowledge and information) | The pharmacists reported working part-time in the clinic even if they wanted to work full-time. They reported that it is related to the low financing accorded by the government for their work. However, some clinics implement compensatory measures to support pharmacists present in the clinic. | - 75% of pharmacists and 85% of other professionals agreed there were sufficient resources to support the intervention. - 55% of pharmacists and 65% of other professionals agreed there was sufficient training to enable staff to implement the intervention. | Overall, the resources available were considered sufficient to support the intervention implementation. Funding compensations were considered helpful for pharmacists, but they are not a viable long-term solution for sustaining their work. To properly implement the intervention, additional training for all team members might be needed. |
| **Outer setting** | | | |
| Policies and laws | The advanced practice partnership agreements were considered useful for empowering pharmacists to make their interventions more easily, even if one pharmacist did not see the worth for her own work.  General practitioners reported trusting the pharmacists with whom they worked because they understood their professional limitations. | - 100% of all participants agreed they were open to working with colleagues in new ways to use the intervention. - 100% of other professionals have confidence in other people’s ability to use the intervention. | Policies favoring pharmacists’ autonomy, such as advanced practice partnership agreements are useful for supporting pharmacists’ participation in the care trajectory and are appreciated by other professionals, who expressed confidence in their colleagues’ ability to use the intervention.  They probably enabled pharmacists to be less reliant on their colleagues, allowing them to better integrate their activities into the trajectory. |
| **Individuals** | | | |
| Innovation deliverers (pharmacists, nurses, general practitioners) | All the professionals reported a strong motivation to implement the pharmacist activities into the trajectory.  The general practitioners considered it useful to collaborate with the pharmacist, as it allowed them to focus on other patients. | - 100% of professionals agreed that their participation in the intervention is a legitimate part of their role. - 75% of pharmacists and 85% of other professionals agreed the work is assigned to those with skills appropriate to the intervention. | The strong motivation of the professionals to include pharmacists in the care trajectory was an important facilitator of the intervention implementation.  Moreover, most professionals considered having the appropriate skills and being legitimate participants in the intervention.  Professionals with high confidence in their capabilities and opportunities are more likely to embrace the changes required to implement pharmacists’ activities in the trajectory. |
| High-level leaders | The pharmacists considered they had enough support from their management (general practitioners in charge), despite isolated case of insufficient support being reported. | - 75% of pharmacists and 85% of other professionals agreed that management adequately supports the intervention | Overall, the support provided by high-level leaders was considered sufficient to enable pharmacists’ participation in the care trajectory. However, when this support is viewed as insufficient, it hinders pharmacists’ ability to fully implement their activities. |
| **Implementation process** | | | |
| Doing | Interviewees reported that the integration of the pharmacists’ activities was reinforced by regular meetings and reminders to the other professionals during the early phases of the implementation process.  They reported that pharmacists had to be proactive in collaborating with other professionals to implement their activities. | - 90% of pharmacists and 80% of professionals agreed there are key people who drive the intervention forward and get others involved. | The pharmacists are key implementation actors. They have to actively promote their participation in the care trajectory and regularly remind their colleagues about their role within the clinic to facilitate intervention implementation. |
| Tailoring strategies | The pharmacists reported modifying the intervention eligibility criteria for patients in response to insufficient funding to maintain a full-time presence. They added more strict referral criteria based on medication. | - 100% of pharmacists and 80% of other professionals agreed they can modify how they work with the intervention. | Tailoring strategies, such as stricter referral criteria, may be needed to facilitate intervention implementation, given limited funding. |
